# Mercury Exposure in Artisanal and Small-Scale Gold Mining Communities in Sukabumi, Indonesia

**DOI:** 10.5696/2156-9614-10.28.201209

**Published:** 2020-12-02

**Authors:** Alfonsus H. Harianja, Grace S. Saragih, Ridwan Fauzi, M. Yusup Hidayat, Yunesfi Syofyan, Ely Rahmy Tapriziah, Sri Endah Kartiningsih

**Affiliations:** Research and Development Center for Environmental Quality and Laboratory (P3KLL), Banten, Indonesia

**Keywords:** ASGM, mercury, perception, socioeconomic characteristics, Sukabumi

## Abstract

**Background.:**

Artisanal and small-scale gold mining (ASGM) is one of the largest sources of mercury (Hg) pollution in Indonesia. In West Java Province, ASGM is found in Bogor, Cianjur, and Sukabumi Regencies.

**Objectives.:**

The present study aimed to evaluate Hg contamination effects and socioeconomic factors in communities living around ASGM operations in Sukabumi Regency.

**Methods.:**

A quantitative method was used to describe socioeconomic ASGM communities. The concentrations of total mercury (T-Hg) in hair were measured in 71 respondents. This study also assessed perception of the use of Hg in the gold ore processing and their impact on the environment.

**Results.:**

The population of gold miners in the studied three villages was 1300 households (25.77% from a total of 5044 households). Artisanal and small-scale gold mining involves both men and women employed as miners and gold amalgam processors, respectively. The average monthly income generated as much as Indonesian Rupiah (IDR) 272 000–2 000 000 (about 19–140 USD). Total Hg analysis was conducted for hair samples of 71 respondents (38 men, 33 women). The results showed an average T-Hg in men of 3.27±2.89 ppm, and women of 5.91±4.69 ppm. The level of T-Hg in the respondents was associated with distance to the ball mills and not related to distance to the mine site.

**Participant Consent.:**

Obtained

**Ethics Approval.:**

This study was approved by Ministry of Environment and Forestry of the Republic of Indonesia

**Competing Interests.:**

The authors declare no competing financial interests.

## Introduction

For more than two decades, artisanal and small-scale gold mining (ASGM) has been operated in Indonesia, spread widely across 31 provinces.^1^ In West Java Province, ASGM is found in Bogor, Cianjur and Sukabumi Regencies. Commonly, ASGM in Indonesia uses mercury (Hg) in gold ore processing.^2^ Mercury is released into the environment during and after this process, and potentially spreads to all environment media, such as air, water, and soil. Studies have found that Hg contamination occurs in water, air, sediments, soil, biotas such as mammals and birds, fishes, rice, vegetables and trees, and miners, processors or other nearby inhabitants to ASGM.^3–16^ Uncontrolled Hg application occurs due to the low technical knowledge of the miners, which makes ASGM the second most significant source of Hg pollution, after coal burning, in Indonesia.^4,17,18^

Elemental Hg and Hg compounds are known to be toxic and can bioaccumulate and be transported long distances. Methyl Hg is formed by oxidation of anaerobic bacteria in sediments and water. It is highly toxic and accumulates in the food chain.^9^ Determination of Hg exposure in the body can be done through the measurement of levels in the body tissues, such as hair, nails and urine which are known as biomarkers.^19,20^ Mercury exposure over a long period of time can trigger health problems in humans, and become highly toxic. Chronic mercury poisoning often occurs in humans who live around ASGM.^21,22^

In the Sukabumi mine, which has been in operation for more than ten years, miners apply Hg in a ball mill gold ore processor.^23^ However, data and information regarding Hg contamination on the environment and in humans at this site are scant. The present study aimed to trace Hg exposure in humans and investigate socioeconomic factors related to Hg exposure in the Sukabumi area. The research findings will be valuable for local and national governments for proposing Hg phase-out in ASGM as part of the National Action plan of Indonesia under the ratification of the Minamata Convention.^24^

## Methods

**Figure 1:**
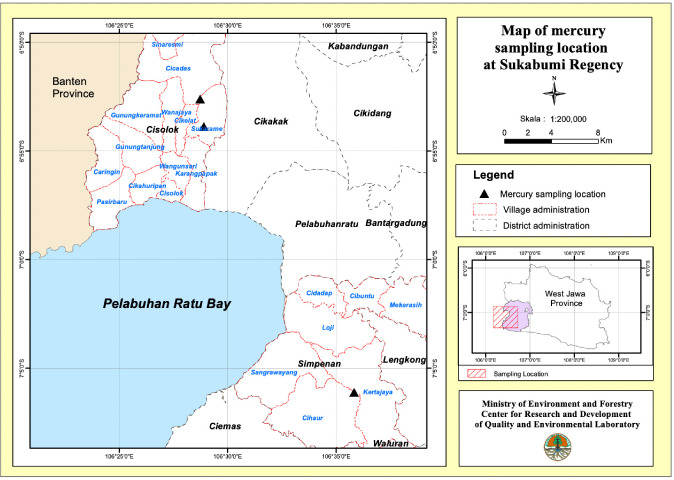
Map of research location

The present study was carried out during February–May 2019 in purposively selected sites in Sukabumi Regency, namely Cicadas village and Sukarame village in the Cisolok sub-district and Kertajaya village in the Simpenan sub-district *(Figure 1).* These locations were chosen because some of the inhabitants in the villages worked as miners or gold ore processors.^9^ All gold mining sites were located in the state forest area, while the gold grinding processors were located in the settlement areas, within 1 to 5 km from the mining sites.

### Data collection procedure

The present study employed a purposive survey method for data collection. Data were gathered through a combination of the quantitative method using structured questionnaires and the qualitative method employing in-depth interviews. A convenience sample of respondents were selected based on their involvement in ASGM or ASGM-related jobs as well as distance from their homes to the ASGM site. All respondents worked in ASGM or ASGM-related jobs, although some worked only occasionally and further identified as ‘housewives'. Subjects consisted of miners and non-miners, such as women involved with gold ore processing, for a total of 71 respondents. Socioeconomic characteristics examined in the survey included age, family size, education level, distance to the location of mining and processing mill, income level, and years living in the village. Additional questions were posed to miners and processors, covering mining processes and gold ore processing procedures, working hours, the use of Hg in the process of ball mill processing, gold ore production, and respondents' history of mining activities. Furthermore, the present study also assessed respondents' knowledge and attitudes towards the use of Hg in gold ore processing and impact on the environment.

**Abbreviations**
*ASGM*: Artisanal and small-scale gold mining
*FAO*: Food and Agriculture Organization
*IDR*: Indonesian Rupiah
*T-Hg*: Total mercury
*USD*: United States Dollars

Hair samples were collected from all 71 respondents. Total mercury (T-Hg) determinations were carried out using a Mercury Analyzer NIC MA-3000 at the Research and Development Center for Environmental Quality and Laboratory in Serpong, Banten Province. The analysis method for T-Hg determination was done using the combustion system, described in the United States Environmental Protection Agency's (USEPA) International Standard 7473.^25^ The accuracy of the analytical method was checked by employing the recovery of Standard Reference Material issued by National Institute of Standards and Technology (SRM NIST 1641d) (the value was 99.9%, recovery of samples spiked with Hg standard was 91.5% to 109.2%) and the blank sample method.^26^ A blank sample was prepared by using a weight of reagent water at the specified weight in the preparation method and then carried through appropriate steps of the analytical process. The result of the blank method was lower than the minimum detection limit (MDL = 0.3 mg/g).

#### Ethics approval

This study was approved by the Ministry of Environment and Forestry of the Republic of Indonesia. The study purpose and protocols were explained, and informed consent was obtained from all respondents prior to interviews and hair sampling.

### Data analyses

The collected data were analyzed using descriptive and statistical analysis. Respondents' socioeconomic background was tabulated and descriptively presented. Respondents' level of knowledge, perception, and attitude toward Hg application in gold ore processing was evaluated using the Likert scoring system. Mercury exposure was measured based on the value of T-Hg in the respondents' hair and then analyzed by comparing those values to the human biomonitoring standard.^27^ This study also examined the effect of socioeconomic factors in Hg exposure in humans using statistical analysis employing the linear regression model.

## Results

Table 1 presents the socioeconomic characteristics of the respondents in the present study. A total of 71 respondents were included in the analysis, with the majority (87.33%) between 20 and 51 years old, locally considered to be of working age. Men worked at the mining site, digging, and transporting materials into the processing facility. Women worked in gold ore grinding facilities, breaking down materials before they are placed on the ball mill.

**Table 1:** Socioeconomic Characteristics of Respondents

**Criteria**	**Classification**	**Number**	**%**
Village	Sukarame	29	40.85
	Cicadas	31	43.66
	Kertajaya	11	15.49
Age (years)	20–27	13	18.31
	28–35	19	26.76
	36–43	14	19.72
	44–51	16	22.54
	52–59	5	7.04
	60–67	2	2.82
	68–75	2	2.82
Gender	Male	38	53.52
	Female	33	46.48
Number of family members	1	1	1.41
	2	6	8.45
	3	25	35.21
	4	25	35.21
	5	10	14.08
	6	4	5.63
Education level	Primary School	50	70.42
	Junior High School	6	8.45
	Senior High School	12	16.90
	Bachelor/University	1	1.41
	N/A	2	2.82
Primary occupation	Civil servant	2	2.82
	Miner	21	29.58
	Farmer	24	33.80
	Private	11	15.49
	Housewife	13	18.31
Income (IDR/month) = x	x≤ 500 000 (USD 34.03)	14	19.72
	500 000 (USD 34.03) < x ≤ 1000.000 (USD 68.09)	22	30.99
	1 000 000 (USD 68.09) < x ≤ 2 000 000 (USD 136.17)	21	29.58
	2 000 000 (USD 136.17) < x ≤ 3 000 000 (USD 204.18)	8	11.27
	x > 3 000 000 (USD 204.18)	4	5.63
	N/A	2	2.82
Water source	River	1	1.41
	Well	22	30.99
	Spring water	37	52.11
	Tap water	11	15.49
Food sources	Traditional market	28	39.44
	Home garden	2	2.82
	Kiosk	41	57.75
Distance of respondents' housing to ball mill	≤ 3 m	34	47.89
	> 3–5 m	l7	23.94
	> 5–10 m	9	12.68
	> 10–20 m	4	5.63
	> 20–50 m	4	5.63
	> 50 m	3	4.23
The distance of respondents’ housing to mine	≤ 1 km	18	25.35
	> 1–2 km	35	49.30
	> 2–5 km	14	19.72
	> 5 km	4	5.63

Abbreviation: USD, United Stated dollar

The majority of respondents attended primary school. Most of the respondents worked as farmers and miners. Some of the residents also work in tea or rubber plantations.^28^ The majority of the subjects lived on a monthly income range of Indonesian Rupiah (IDR) 500,000–1,000,000 (USD 34.03–USD 68.09). Compared to the Food and Agriculture Organization (FAO) standard, the majority were living below the poverty line.^29^ Poverty has also been documented at other ASGM communities, such as Sumbawa, where ASGM has not boosted the residents' income.^30^ Most of the respondents stated that the mining site was located 0.5 km-1.5 km from their residence and the ball mill processing was located in the backyard or beside their houses.

**Figure 2:**
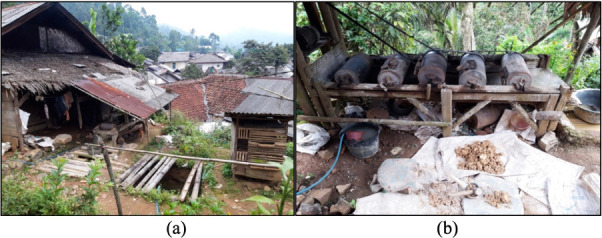
Gold ball mill in Lebak Nangka sub-village, Sukarame sub-district, Sukabumi Regency. (a) Settlement (b) Ball mill facility

Figure 2 shows the settlements and a ball mill facility in the study area.

The present study evaluated the perception and knowledge of respondents towards Hg usage and the potential of Hg residue to contaminate neighborhoods near AGSM activities. On a total of ten questions, respondents' answers were scored on a 5-point Likert scale (strongly disagree, disagree, unsure, agree, and strongly agree) and the results are presented in Table 2.

**Table 2:** Respondents' Perception and Knowledge of Mercury Usage in ASGM

**Statement**	**Score**	**Criteria**	**Percentage (%)**
The use of mercury is very harmful to the health of gold miners and the surrounding community	66.76	Agree	46.91
Mercury pollution in the air, soil, and water can interfere with human health and the environment	68.17	Agree	48.15
The handling of mercury pollution in the environment is crucial	69.01	Agree	40.74
The use of mercury in gold processing should be limited or reduced	63.38	Agree	38.27
Gold processing should be done without mercury	62.25	Agree	50.62
Ball mill gold ore processing should be located far away from settlements	68.17	Agree	45.68
The community should reduce mercury use	58.87	Unsure	35.80
Reducing mercury pollution should involve communities and local governments	69.30	Agree	41.98
Alternative sources of income replacing ASGM are available	59.72	Unsure	44.44
Alternative livelihood should provide higher incomes	60.56	Agree	43.21

Remarks, range value for criteria: 0–20 (strongly disagree); 21–40 (disagree); 41–60 (unsure); 61–80 (agree); 81–100 (strongly agree).

As seen in Table 2, most of the respondents in the research area indicated that they are aware of the dangers of using Hg in the gold amalgamation process. They also agreed with the statement that Hg is very harmful to human health. Most respondents agreed with the statement that Hg pollution in the air, soil, and water can pose a risk to human health. Related to the handling of Hg pollution, most respondents agreed with the statement that the handling of Hg pollution is crucial. Most of the subjects suggested limiting Hg usage in the gold ore process or phasing it out. This is in line with the government decision to reduce or phase out Hg in ASGM in Indonesia by 2030, as stated in Presidential Decree Number 21/2019^24^ supporting non-mercury gold processing technologies.^31^ The respondents also agreed with the statement that ASGM facilities should be located outside of settlement areas to minimize their contact with Hg. They believe that the process of Hg reduction or phase-out in ASGM should be done by the community in collaboration with local governments, but they were unsure if the process would be a success if performed without assistance. They were also unsure about available alternative sources of income. They also pointed to the need for alternative occupations providing a higher income than ASGM.

Hair is a preferred biomarker because of its simple collection technique, storage, and analysis.^32^ The results of T-Hg in the respondents' hair samples is presented in Table 3. Hair Hg levels from three ASGM communities ranged from 0.71 to 24 ppm and averaged 4.34 ppm.

**Table 3:** Mercury Content in Hair Samples

**Group**	**No.**	**Age (year)**	**Hg range (ppm)**	**Hg average ± SD (ppm)**
Males	48	23–70	0.71–18	3.27±2.89
Females	33	22–60	1.5–24	5.91±4.69
Children	4	2.5 – 10	0.96–8.1	5.34

**Figure 3:**
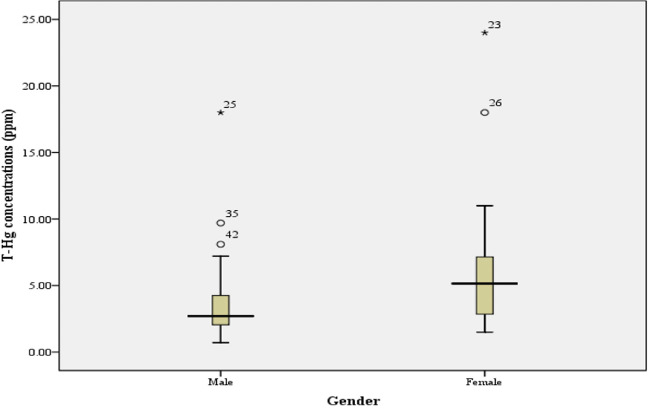
Box plot representing the concentration of T-Hg in in hair of male and female respondents, outliers (o), and extremes (*).

The present study found a significant difference between Hg in hair of males and females (*P* = 0.02, *P* < 0.05), as presented in Figure 3. The average Hg content was higher in females.

The classification of the risk level of T-Hg found in human hair is displayed in Table 4. The level of Hg content in respondents' hair in the research area can be classified into the alert and high-level categories in terms of human biomonitoring classification.^27,33,34^ Only three males had Hg content at a normal level. Mercury content in females were classified in the alert and high levels. Possible explanations for these results were analyzed using explanatory variables such as socioeconomic characteristics.

**Table 4:** Classification of Mercury Content in Hair

*Toxicology threshold limit	**Category**	**Number of samples**	

		**Men**	**Women**
<1 μg Hg/g hair	Normal	2	
1-<5 μg Hg/g hair	Alert level	28	16
≥5 μg Hg/g hair	High level	8	17

^*^Human biomonitoring categories ^27,33,34^

Table 5 presents the result of the multiple linear regression, and the tested independent factors affecting T-Hg contamination in respondents' hair. The factors predicted to affect the levels of mercury in hair (Y_i_) included age (X_1_), number of family members (X_2_), education level (X_3_), income level (X_4_), length of stay at the location (X_5_), distance from the respondent's house to the mining sites (X_6_), distance of the respondent's house to the ball mill location (X_7_), and perception (X_8_). The results indicated that the three factors affecting the levels of Hg in hair were education level (X_3_), distance from residence to mining sites (X_6_), and the distance from residence to the ball mill (X_7_). The regression model's ability to predict the effect of social-economic factors affecting Hg levels in hair is qualified by *P*-value < 0.001 and R^2^ 0.36. The regression model selected in the analysis was a robust regression model to eliminate the problem of possible homoscedasticity.^35^

**Table 5:** Multiple Linear Regression Results

**Variables**	**Coefficient (SE)**	*P***-value**
Age (X_1_)	−0.082 (0.056)	0.15
Number of family members (X_2_)	−0.060 (0.251)	0.81
Education (X_3_)	−1.188 (0.459)	<0.01
Income (X_4_)	0.000 (0.000)	0.27
Length of stay (X_5_)	−0.008 (0.043)	0.86
Distance to mining site (X_6_)	−0.001 (0.000)	<0.01
Distance to ball mill (X_7_)	0.100 (0.009)	<0.01
Knowledge (X_8_)	0.063 (0.070)	0.38

## Discussion

There are several gold ore processing locations in Cicadas Village, although there is no gold mining sites in this area. The villagers received material containing gold ore from the neighboring village, Sukarame. Cicadas villagers process the material containing gold using a ball mill. Based on an interview with village government officials, about 500 households (of a population of 1 751 individuals) are involved in ASGM activities in Cicadas.

One of the ball mill owners explained that his business was started around ten years ago, with an initial capital of IDR 50,000,000 (USD 3402.78) He bought the material from miners in Sukarame and the price ranged from IDR 50,000-100,000 (USD 3.4 – 6.8) per sack of ore (±50 kg), depending on the predicted quality and the deal between the ball mill owner and the miners. If the ball mills owners also manage the mining operation, then they can employ up to 50 miners. They share the ore containing gold materials equally.

The leader of Sukarame Village indicated that the ASGM has been present since the 1990s. Many inhabitants perform mining extraction in the state-owned forest and the gold processing in their settlement area. These activities are illegal, and therefore there are no official records of gold miners. Around 35% of the total households are miners. Women are involved in breaking down materials and can process as many as five sacks of material each day. In comparison, men can process seven sacks daily. Mercury is used in ball mill grinding tubes to bind gold ores, with as much as 500 g of Hg used for each ball mill per week. Based on field observation, there are hundreds of ball mills in Sukarame village *(Figure 2)* which operate 24 hours, seven days a week.

A similar system also applies to Kertajaya Village, which has been in operation since the Dutch colonial era. The villagers have been working in ASGM for more than 50 years. Based on the information gathered from a ball mill owner, there are approximately 500 households involved in the business. The laborers are paid IDR 7 (approximately USD 0.5) per sack. This job can be done by men and women to obtain additional income.

Mercury usage ranged from 1–4 oz to produce 2.4–4 grams of gold per month on average in Cicadas and Sukarame Villages. Since the total number of ASGM in these villages is 1.3 units, the average use of Hg in gold amalgamation ranged from 260–520 kg/month, which is eventually released as a byproduct into the environment. This is lower than the amounts of Hg used in other areas such as Poboya and Sekotong.^31^

In the present study, an elevated Hg level of 24 ppm was found in a 23-year-old woman. This was a result of the addition of Hg in the ball mill. During this process, workers do not wear masks or gloves and thus have direct contact with Hg. Another associated factor is the process of releasing the gold from the amalgamation by burning the gold-mercury compound.^2,36^ Local residents are exposed to Hg vapor through inhalation from the amalgam burning that are located on or nearby the settlements.

The results showed that the average Hg content was higher in females than males. However, some factors related to hair Hg concentration were not considered in the present study, including dietary habits and water consumption. In comparison, the total Hg concentrations in hair of people directly exposed to Hg in Bombana, Southeast Sulawesi ranged from 3.29 to 81.44 ppm.^33^ Meanwhile, in North Gorontalo Regency, the highest Hg level of 17.9 ppm was found in the miner group.^34^

The education level of respondents had significant effects (p < 0.01) on hair Hg content. Respondents with higher education tended to have lower Hg levels. The distance between the house and the mine hole showed a negative relationship, thus the farther from the mine, the lower the T-Hg in hair. Different results were found for the distance of the house to the ball mills. The results showed a positive relationship, thus the closer to the ball mill, the lower the T-Hg in hair*.* The distance of the ball mills from the inhabitants' residence ranged from 1–150 meters, which suggests that the T-Hg in hair does not come from ball mills, but from mercury-gold amalgam burning activities. Mercury burning is mostly carried out in gold collectors' houses which are located within the same area as the ball mills. This phenomenon is possibly related to the Hg released into the air through the combustion chamber during the gold purification process.

Other studies in Indonesia also found hair Hg contamination. In Gorontalo Utara Regency, Gorontalo Province, the hair Hg levels of ASGM miners ranged from 7.1–17.9 ppm.^34^ The mean hair Hg levels of miners and non-miners in Sekotong ASGM were 2.77±1.68 ppm and 2.37±1.82 ppm, respectively, and the highest level of Hg in hair of miners was 12.93 ppm.^37^ Another study reported that mercury content in hair sampled in an ASGM community in Cisitu, Banten province reached as high as 25 ppm.^22^

Mercury exposure to humans can originate from skin lightening products, dental amalgam filling, consumption of Hg-contaminated fish, vegetables, rice, and drinking water.^14,38–46^ People who live and work at or near ASGM areas are vulnerable to health issues caused by Hg vapor inhalation such as neurological, digestive, and immune system disorders.^47^ Some symptoms of mercury intoxication are sleeping troubles, tremors, numbness, and headaches.^22^ Inorganic Hg accumulates in the kidneys and induces kidney damage.^42^

A limitation of the present study was the lack of examination of Hg intoxication symptoms experienced by participants. However, few adults and children in the research with high T-Hg levels showed neurological symptoms. This could be related to Hg contamination in this area, as suggested by a previous study.^48^

A reduction or phase-out of mercury application in ASGM is necessary based on the results of the community evaluation.^49^ However, government support for these measures is crucial, as has been proposed in Nigeria, especially in providing new employment alternatives that could replace gold mining activities.^50,51^ As these villages are located near the state forest, the government could introduce a social forestry management system as an alternate source of income based on economic and ecological feasibility.^52–54^

## Conclusions

The present study found that inhabitants living around ASGM had elevated hair Hg content, with the majority classified in alert and high levels. Women had higher Hg content than men. Factors affecting the level of Hg content in hair included education level and distance of the mining site and ball mills to settlements. The respondents were familiar with steps to minimize the health effects of ASGM and agreed to relocate ball mills away from their residences. This research recommends several actions: providing alternative livelihoods that create higher income than gold mining; developing social forestry programs; conducting further health examinations on those with high Hg levels; and increasing public knowledge of the dangers of Hg on the environment and public health.
